# Maintenance of Leptospira Species in Leptospira Vanaporn Wuthiekanun Agar

**DOI:** 10.1128/JCM.02273-14

**Published:** 2014-12

**Authors:** Vanaporn Wuthiekanun, Premjit Amornchai, Sayan Langla, Malinee Oyuchua, Nicholas P. J. Day, Direk Limmathurotsakul

**Affiliations:** a Mahidol-Oxford Tropical Medicine Research Unit, Faculty of Tropical Medicine, Mahidol University, Bangkok, Thailand; b Nuffield Department of Medicine, University of Oxford, Old Road Campus, Headington, Oxford, United Kingdom; c Department of Tropical Hygiene, Faculty of Tropical Medicine, Mahidol University, Bangkok, Thailand

## Abstract

The maintenance of Leptospira species in liquid or semisolid medium is time-consuming and at risk of contamination due to the needs of routine subculture and dark field microscopy. Using Leptospira Vanaporn Wuthiekanun (LVW) agar, we maintained 100 pathogenic Leptospira isolates for 12 months without the need for subculture and confirmed the viability of all isolates by the naked eye.

## TEXT

Leptospirosis is a zoonotic disease caused by pathogenic members of the genus Leptospira (1, 2), and the ability to isolate and maintain Leptospira spp. is critical for both diagnostic and research purposes. The isolation of Leptospira spp. from clinical specimens and the microscopic agglutination test (MAT) are commonly used as reference tests for the diagnosis of leptospirosis (3). Leptospira spp. do not grow on conventional media. Isolation of this organism from clinical specimens usually requires a specific medium (for example, Ellinghausen and McCullough modified Johnson and Harris [EMJH] liquid medium containing 3% rabbit serum, and semisolid EMJH medium containing 3% rabbit serum and 0.1% bacteriological agar) and considerable expertise, and it takes between 30 and 90 days (4). Genotyping and serovar identification of the isolated organism play important roles in a research setting in the understanding of its epidemiology (5, 6). MAT is a serological test detecting antibodies against Leptospira organisms, and it requires a range of live organisms with representative serovars known to cause leptospirosis in a study region (3).

In general, Leptospira isolates are maintained at room temperature in liquid or semisolid medium, with frequent transfer into fresh medium required every 3 to 12 weeks. The viabilities of the isolates are then confirmed by dark-field microscopy (7). This preservation method is labor-intensive and at high risk for contamination. Liquid nitrogen freezing is recommended for long-term preservation of Leptospira spp. isolates (8); however, this procedure requires expensive facilities that are usually unaffordable for most laboratories in developing countries where the disease is endemic. We recently described a new solid agar called Leptospira Vanaporn Wuthiekanun (LVW) agar, which enables rapid growth, the isolation of single colonies, and simple antimicrobial susceptibility testing for Leptospira spp. (9). Here, we describe a simple method using LVW agar to maintain pathogenic Leptospira isolates at room temperature for ≥12 months without the need for a medium transfer, and with the added advantage that the confirmation of organism viability can be carried out by the naked eye.

In this study, a total of 100 pathogenic human Leptospira isolates of known serovars were used in this study. This collection of Leptospira spp. consisted of human isolates from Thailand (*n* = 65), Laos (*n* = 26), and Sri Lanka (*n* = 9). There were four species included: Leptospira interrogans (*n* = 90), Leptospira borgpetersenii (*n* = 4), Leptospira kirschneri (*n* = 3), and Leptospira weilii (*n* = 3), which were additionally classified into 11 serovars (Autumnalis [*n* = 54], Pyrogenes [*n* = 13], Grippotyphosa [*n* = 7], Bataviae [*n* = 5], Canicola [*n* = 5], Javanica [*n* = 4], Medanensis [*n* = 3], Mengdeng [*n* = 3], Hebdomadis [*n* = 2], Pomona [*n* = 2], and Wolffi [*n* = 2]). All organisms were maintained in fresh semisolid EMJH medium containing 3% rabbit serum and 0.1% bacteriological agar. A transfer into fresh medium was performed every 3 months (stock culture).

LVW agar was made as previously described (9). In short, for 1 liter of LVW agar, 800 ml of water, 10 g of Noble agar base (Becton Dickinson), 10 mg of sodium pyruvate (Merck), and 2.3 g of Leptospira medium base EMJH (Difco) were mixed and autoclaved at 121°C for 20 min. Next, 100 ml of Leptospira enrichment EMJH (Difco) and 100 ml of heat-inactivated rabbit serum (Gibco) were added when the autoclaved component was at 50°C. After that, 3 ml of mixed LVW agar was poured into a 5-ml plain sterile blood collection tube (catalog no. Z6744; Teklab, County Durham, United Kingdom). After the inoculation of 100 μl of Leptospira isolate from stock culture (approximately 10^6^ to 10^7^ CFU/ml), the tube caps were fully tightened and then twisted back for half a turn. The tubes were stored at room temperature (25 to 30°C) in air and were observed by the naked eye for the presence of subsurface Leptospira bands weekly during the first month and then monthly for a total of 12 months. At the end of the study, the viabilities of the Leptospira isolates were determined by two methods, (i) dark-field microscopy and (ii) colony observation. Colony observation was performed by dropping 10 μl of the supernatant of the study tubes on an LVW agar plate, which was incubated at 30°C in CO_2_ for 2 days and then in air for a total of 28 days to observe the growth of Leptospira colonies, as previously described (9).

**FIG 1:**
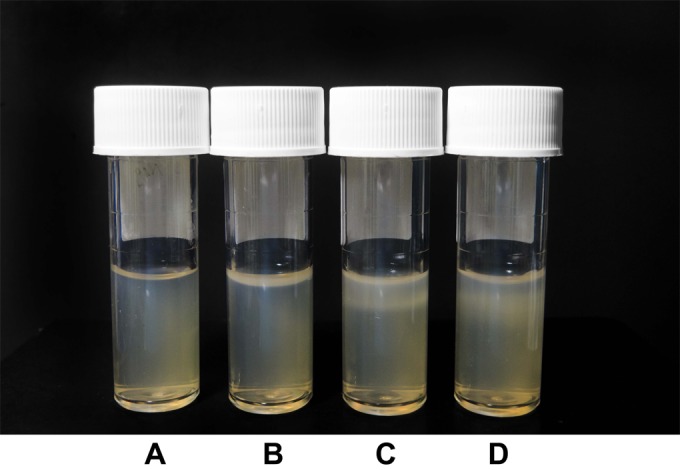
The subsurface Leptospira band of L. interrogans serovar Pyrogenes (strain R601) after inoculation into LVW solid agar compared to that with a fresh LVW solid agar tube (tube A). Tubes B, C, and D show the Leptospira bands observed at 1 week, 3 weeks, and 2 months after inoculation, respectively. Tubes B and C have subsurface Leptospira band thicknesses of 2 mm and 7 mm, respectively. In tube D, the subsurface Leptospira band has dispersed to the bottom of the tube, the edge of the band could not be clearly determined, and the thickness could not be measured.

Of 100 pathogenic Leptospira isolates, 85 developed the subsurface Leptospira band in LVW agar at 1 week after inoculation (median band thickness, 1 mm; interquartile range [IQR], 0.5 to 3 mm; range, 0 to 9 mm) (Fig. 1; see also the supplemental material). At 2 weeks after inoculation, another 13 isolates formed the band, and the median thickness of the band increased significantly to 4 mm (IQR, 3 to 5 mm; range, 0 to 17 mm; *P* < 0.001, Wilcoxon signed-rank test). At 3 weeks after inoculation, all 100 isolates showed the subsurface Leptospira band in LVW agar, with a median thickness of 6 mm (IQR, 4 to 6 mm; range, 0.5 to 17 mm).

At 2 months after inoculation, the subsurface Leptospira band of 4 isolates (4%) dispersed to the bottom of the tube, the tube was turbid, and the edge of the band could not be clearly determined. At 3 and 6 months after inoculation, the dispersion of the subsurface Leptospira band was observed in a total of 12 and 40 isolates (12% and 40%), respectively. At 12 months after inoculation, the dispersion of the subsurface Leptospira band was observed in 54 isolates (54%). There was evidence suggesting that the likelihood of dispersion observed at month 12 differed between serovars (*P* = 0.01, Fisher's exact test; Table 1). For example, the dispersion was observed in 41% (22/54) of the Leptospira serovar Autumnalis isolates compared to 92% (12/13) of the Leptospira serovar Pyrogenes isolates.

**TABLE 1:** Dispersion of the subsurface Leptospira band 12 months after inoculation into LVW agar by species and serovar^*a*^

Species	Serovar	% dispersion at 12 mo (no. dispersed/total no.)
L. interrogans	Autumnalis	41 (22/54)
	Pyrogenes	92 (12/13)
	Bataviae	80 (4/5)
	Canicola	40 (2/5)
	Grippotyphosa	50 (2/4)
	Medanensis	33 (1/3)
	Hebdomadis	50 (1/2)
	Pomona	50 (1/2)
	Wolffi	100 (2/2)
L. borgpetersenii	Javanica	50 (2/4)
L. kirschneri	Grippotyphosa	67 (2/3)
L. weilii	Mengdeng	100 (3/3)

a *n* = 100 isolates.

At 12 months after inoculation, there was no change in the volume of agar, but dryness of the agar was observed in all tubes. Therefore, 700 μl of EMJH broth containing 3% rabbit serum was added to each tube, and the viabilities of all Leptospira isolates were evaluated 1 week later. On dark-field microscopy, the number of Leptospira organisms was detected to be approximately 10^7^ to 10^8^ cells/ml, with 100% active motility, for all isolates. After dropping 10 μl of the supernatant of the study tubes on LVW agar plates and incubating the agar plates for 1 week, subsurface colonies of Leptospira were observed for 99 isolates (99%). Subsurface colonies of the remaining strain of L. weilii serovar Mengdeng (NR-20182) were observed at 2 weeks after the incubation. There were 3 isolates (3%) that grew Leptospira spp. with contaminated coagulase-negative Staphylococcus. The separation of Leptospira isolates from the bacterial contamination was successfully achieved by colony selection in all 3 cases.

In this study, we developed a simple protocol for using LVW agar to maintain Leptospira species. This method is simple, inexpensive, and less time- and labor-consuming than the conventional maintenance of Leptospira spp. in liquid or semisolid medium (7). Routine subculture is not required in this simple protocol using LVW agar. To our knowledge, the contamination rate observed in our study (3% over 1 year) is much lower than that observed in some laboratories using the conventional method with routine subculture. For the contaminated isolates in our study, subsurface colonies of Leptospira on LVW agar were easily separated and purified. As this new method does not require frequent medium transfer, the chance of strain switching or tube mislabeling, which are other problems of long-term maintenance of Leptospira isolates (10), is low. The ability to detect the viability of Leptospira by colony observation and the naked eye without the need for dark-field microscopy will allow this method to be used in resource-limited settings. Factors associated with the dispersion of the subsurface Leptospira band in LVW agar and its association with serovar will need further study.

## Supplementary Material

Supplemental material
