# Supplementary material for: Maintenance of Leptospira Species in Leptospira Vanaporn Wuthiekanun Agar
Source: J Clin Microbiol. 2014 Dec;52(12):4350–2. doi: 10.1128/JCM.02273-14 (PMC4313312; doi:10.1128/JCM.02273-14)
Supplement: Supplemental material [file supp_52_12_4350__index.html]

Maintenance of Leptospira Species in Leptospira Vanaporn Wuthiekanun Agar — Supplemental material 

# Maintenance of Leptospira Species in Leptospira Vanaporn Wuthiekanun Agar

## Supplemental material

**Files in this Data Supplement:**

- Supplemental file 1 -

  Dataset S1 (List of the *Leptospira* species isolates and thickness of the subsurface *Leptospira* band observed over the study period)

  XLS, 54K
